# Deficient Audiovisual Speech Perception in Schizophrenia: An ERP Study

**DOI:** 10.3390/brainsci13060970

**Published:** 2023-06-19

**Authors:** Erfan Ghaneirad, Ellyn Saenger, Gregor R. Szycik, Anja Čuš, Laura Möde, Christopher Sinke, Daniel Wiswede, Stefan Bleich, Anna Borgolte

**Affiliations:** 1 Department of Psychiatry, Social Psychiatry and Psychotherapy, Hannover Medical School, 30635 Hanover, Germany; saenger.ellyn@mh-hannover.de (E.S.); szycik.gregor@mh-hannover.de (G.R.S.); cus.anja@mh-hannover.de (A.Č.); moede.laura@mh-hannover.de (L.M.); sinke.christopher@mh-hannover.de (C.S.); bleich.stefan@mh-hannover.de (S.B.); borgolte.anna@mh-hannover.de (A.B.); 2 Department of Neurology, University of Lübeck, 23562 Lübeck, Germany; daniel.wiswede@neuro.uni-luebeck.de; 3 Center for Systems Neuroscience, University of Veterinary Medicine, 30559 Hanover, Germany

**Keywords:** schizophrenia, multisensory speech perception, audiovisual speech, early sensory processing of speech, EEG

## Abstract

In everyday verbal communication, auditory speech perception is often disturbed by background noise. Especially in disadvantageous hearing conditions, additional visual articulatory information (e.g., lip movement) can positively contribute to speech comprehension. Patients with schizophrenia (SZs) demonstrate an aberrant ability to integrate visual and auditory sensory input during speech perception. Current findings about underlying neural mechanisms of this deficit are inconsistent. Particularly and despite the importance of early sensory processing in speech perception, very few studies have addressed these processes in SZs. Thus, in the present study, we examined 20 adult subjects with SZ and 21 healthy controls (HCs) while presenting audiovisual spoken words (disyllabic nouns) either superimposed by white noise (−12 dB signal-to-noise ratio) or not. In addition to behavioral data, event-related brain potentials (ERPs) were recorded. Our results demonstrate reduced speech comprehension for SZs compared to HCs under noisy conditions. Moreover, we found altered N1 amplitudes in SZ during speech perception, while P2 amplitudes and the N1-P2 complex were similar to HCs, indicating that there may be disturbances in multimodal speech perception at an early stage of processing, which may be due to deficits in auditory speech perception. Moreover, a positive relationship between fronto-central N1 amplitudes and the positive subscale of the Positive and Negative Syndrome Scale (PANSS) has been observed.

## 1. Introduction

Patients with schizophrenia (SZs) not only suffer from characteristic positive and negative symptoms, but also from significant social deficits and consequent social isolation, which negatively influences the course of disease and leads to a reduced quality of life [1,2]. Among other cognitive and emotional functions that determine social competency, speech perception plays an important role in social communication [3] and has been shown to be altered in individuals suffering from SZ [4,5,6].

Contrary to popular belief, speech perception in real life does not exclusively rely on auditory sensory inputs, but also on visual cues, such as the speaker’s articulatory movements [7,8]. During speech comprehension, visual stimuli gain importance when the auditory signal is ambiguous due to adverse listening conditions, such as noisy environments [9]. Therefore, in everyday situations, a sufficient ability to integrate information conveyed from multiple senses is important for speech perception [10,11].

Binding sensory inputs from multiple modalities to form a coherent percept requires coordinated processes engaging different cortical areas, which are presumed to be impaired in SZs according to current pathophysiological theories of schizophrenia [12,13]. Indeed, recent evidence demonstrates that schizophrenia has been associated with abnormalities in multisensory integration (MSI) [14,15,16], as well as impairments in perceptual processes that are crucial for multisensory integration, including temporal processing [17,18,19]. For instance, in a widely used multisensory speech paradigm, known as McGurk illusion [7], SZs showed fewer illusions, indicating reduced cross-modal effects of visual-articulatory signals on auditory speech perception [20,21,22,23]. In the McGurk task, the simultaneous presentation of a video of a face articulating a syllable (e.g., /ga/) and an incongruent auditory sound (e.g., /ba/) causes an illusionary fused percept, different from both the visual and the auditory cue (e.g., “da”). However, it should be noted that two other studies [18,24] did not replicate the reduced occurrence of illusions in SZs. However, both of these studies additionally examined the temporal domain of audiovisual speech perception and reported a prolonged window of perceived simultaneity of asynchronous audiovisual syllables in SZs compared to HCs.

Abnormalities in neural correlates underlying audiovisual speech perception in SZs have been primarily reported in the superior temporal sulcus (STS; [25,26,27]), inferior frontal gyrus (IFG; [25,26,27,28]) and fusiform gyrus [26,28]. Despite intact performance in the McGurk paradigm, Roa Romero et al. [24] found aberrant short-latency and larger-latency alpha band EEG oscillation in SZs. Studies with healthy participants have linked the amplitudes of the early auditory evoked N1 and P2 components of event-related potentials (ERPs) to audiovisual speech processing [29,30,31,32]. In particular, a reduction of the N1 component represents an early multisensory facilitation of auditory speech processing [29,33]. The auditory-evoked P2 has been found to be more negative for incongruent audiovisual pairings (e.g., hearing /ba/ while lip-reading /fu/) than congruent ones [3].

To the best of our knowledge, only two studies have investigated early components of speech perception in SZs. Stekelenburg et al. [34] compared the N1/P2 suppression effect in SZs and HCs for auditory vs. audiovisual speech stimuli. They reported a lack of audiovisual N1 suppression and a reduced congruency effect of the P2 amplitude in SZs. In a recent study, Senkowski and Moran [35] presented audiovisual syllables at three different noise levels (no noise, low noise and high noise). They found that SZs showed reduced performance in speech recognition in the no-noise condition, while the performance differences were diminished or absent in the low- and high-noise conditions, respectively. These behavioral differences were accompanied by a significantly reduced N1 amplitude in SZs compared to HCs in the no-noise condition, while no group difference was observed in the low- and high-noise conditions. The authors suggest that the observed auditory and audiovisual speech recognition deficits in SZs are primarily due to abnormal auditory syllable processing. This implies that SZs have a relatively intact multisensory gain [35].

The present study aimed to provide a more accurate understanding of the role of early neural processing in audiovisual speech perception in SZs by utilizing a naturalistic whole-word speech-in-noise paradigm. In an ERP experiment, audiovisual speech stimuli, including both the voice and lip movements, were superimposed by white noise and presented to participants with SZs and HCs. In line with previous research, we anticipated deficits in multisensory speech perception in SZs compared to HCs. Moreover, we hypothesized group differences in early ERP components associated with the audiovisual (AV) task demands.

## 2. Materials and Methods

### 2.1. Participants

A total of 20 individuals diagnosed with schizophrenia spectrum disorders (8 women, age 41.9 ± 13.8) and 21 matched healthy controls (9 women, mean age = 38.2 ± 13.3) participated in the current study. Patients were recruited from the psychiatric department of Hannover Medical School and were diagnosed by experienced physicians based on DSM-IV-TR [36]. The German adaptation of the Positive and Negative Syndrome Scale (PANSS) for schizophrenia [37] was used to assess positive and negative symptoms via interviews. Participants in the control group had not experienced any substantial psychiatric disorder in the past. Participants with acute drug or alcohol abuse, as well as those with acute psychotic symptoms, were excluded from the study. All patients received atypical antipsychotic medication. The average duration of their disorder was 13.9 ± 10.2 years.

The healthy controls were matched for age and sex. Verbal IQ was assessed using the MWT-B [38]. All subjects had normal or corrected-to-normal vision and were native speakers of German (see Table 1 for sociodemographic characteristic, cognitive performance and patients’ clinical scores). The study was approved by the ethics committee of Hannover Medical School and all participants provided written informed consent.

### 2.2. Stimuli and Paradigm

The participants were instructed to complete an adapted version of the speech perception task that was previously employed by Ross et al. [39]. This adaptation has been successfully used and evaluated in our lab [40,41]. The stimuli were selected from the German part of the CELEX database [42] with a Mannheim frequency of 1,000,000 (MannMln) of at least one. This frequency indicates the occurrence of the selected word per 1 million words taken from the Mannheim 6.0-million-word corpus. Visual stimuli were videos with a duration of 2 s showing a male native German speaker with linguistic experience pronouncing a single word.

The videos displayed a frontal view of the speaker’s entire face (720 × 576-pixel resolution, covering 25° vertically and 20° horizontally of the visual angle). A total of 140 words were presented to the participants in a randomized manner, selected from a pool of 400 dissyllabic nouns. The videos were accompanied by audio streams in mono mode presented through two speakers positioned on the left and right side of the video monitor [21′ Sony Trinitron Multiscan G520 (Sony Electronics Inc., San Diego, CA, USA) with 1024 × 768-pixel resolution].

**Figure 1:**
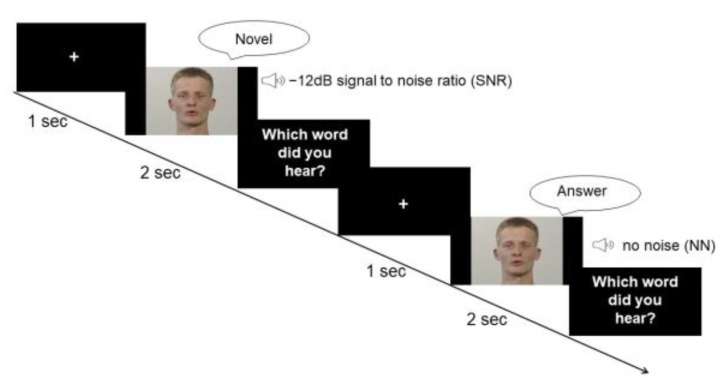
Paradigm. Participants were provided with visual information about the lip movement and auditory information of the voice of a male speaker who pronounced disyllabic nouns from the CELEX database. In the first condition, the speaker’s voice was superimposed by white noise with a SNR of −12 dB. The second condition did not involve any background noise.

The task comprised a condition without white noise (no noise, NN) and one condition with white noise blended into the audio stream (speaker’s voice). The loudness level of white noise was adapted to drown the speaker’s voice with a signal-to-noise ratio (SNR) of −12 dB. According to previous studies, the largest multisensory gain in speech comprehension occurs at a SNR of −12 dB [9,39,43]. Thus, our study design focused on a −12 dB condition, which consisted of 80 trials. Due to time and economic reasons, as well as considering the anticipated small variability in the data for the NN, the trial number for the NN was reduced to 20 trials. Nonetheless, in order to prevent a habituation effect to the −12 dB condition, two other SNRs of −16 dB and −8 dB were also included for 20 trials each. The experiment was performed using Presentation^®^ software (Version 17.2, Neurobehavioral Systems, Inc., Berkeley, CA, USA). The stimuli were presented in a randomized order. Each trial started with a fixation cross of a 1 s duration. During the experiment, participants were instructed to watch the screen and listen to the voices (see Figure 1).

Following each trial, participants were asked to verbally report which word they perceived. They were instructed to guess the answer or report “I did not understand anything” if they had not clearly understood the word. The experimenter recorded the answer without giving any feedback. Any response that did not match the presented word was considered as false. The experimenter started the next trial after receiving the response.

### 2.3. EEG Acquisition and Processing

While participants performed the speech-in-noise-task, EEG was recorded from 32 Ag/AgCl electrodes according to the 10/10 system using active electrodes in an elastic cap. Sampling rate was 512 Hz and impedances were kept below 25 Ω. A BioSemi active electrode system (BioSemi B.V., Amsterdam, Netherlands) and the accompanying ActiView software package were used. The BioSemi system uses a CMS/DLR feedback loop instead of reference and ground with two additional electrodes. To control for eye movement artifacts, no additional electrodes were needed, since the analysis software uses an internal model of eye artifact topographies to control for eye movement artifacts.

EEG data processing was performed by BESA research 6.0 (BESA GmbH, Graefelfing, Germany). Raw data were filtered with a 0.1 Hz high-pass filter, a 30 Hz low-pass filter and a 50 Hz notch filter. Blink artifacts were corrected using an internal model of eye artifact topographies as implemented in BESA software using the virtually created vertical and horizontal electrooculogram channels. Data were segmented in event-related epochs of 1100 ms with a baseline starting 100 ms before stimulus onset. Baseline correction and automatic artifact rejection were performed (maximal amplitudes of 120 µV and maximal difference values of 75 µV). Individual and grand averages were generated for each SNR condition separately for both groups.

In order to establish the times of interest (TOI), we calculated an ERP grand average over groups and conditions. We observed the first negative peak (N1) at 85 ms, accompanied by a subsequent positive P2 peak at 175 ms after stimulus onset. Consequently, we defined the TOI for the statistical analysis of the N1 component as the time window ranging from 60 to 110 ms. Additionally, for the broader P2 component, we defined the TOI as the interval spanning from 140 to 210 ms. To define the region of interest (ROI) and based on previous studies [29,32,34,35,43], the frontal, fronto-central, central and central–partial (F3, Fz, F4, Fc1, Fc2, C3, Cz, C4, Cp1, Cp2) electrodes were incorporated into a mass univariate analysis [44] using MATLAB software (Version R2023a, The MathWorks Inc., Natick, MA, USA). We controlled for familywise error rates by means of permutation thresholding (2500 permutations) and the alpha level was set to 0.05 (two-tailed). The electrodes showing the most robust effects and consistent patterns of activation were chosen for subsequent analysis. The selected electrodes were Fz, Fc1, Fc2 and Cz.

### 2.4. Data Analysis

All statistical analyses were performed with IBM SPSS 28 (IBM Corporation, Armonk, NY, USA). Demographic differences between groups were assessed with independent-sample *t*-tests for continuous variables and chi-square statistic for categorical variables. To assess differences in speech comprehension between SZ and HC, a 2 × 2 repeated-measures analysis of variance (ANOVA) was performed, with SNR (NN vs. −12 dB) as the within-subjects factor and group (SZ vs. HC) as the between-subjects factor.

The evaluation of electrophysiological data included the analysis of the N1 and the P2 components separately. Accordingly, two distinct 2 × 2 × 4 repeated-measures ANOVAs were computed with SNR (NN vs. −12 dB) and electrode (Fc1, Fz, Fc2 and Cz) as the within-subjects factors and group (SZs vs. HCs) as the between-subjects factor. The respective ANOVA was calculated for the N1-P2 complex as well, which was defined as the difference between the amplitude peaks of N1 and P2. When a significant group difference in ERPs was observed, a factorial ANCOVA was performed to control for the potential effect of IQ by including electrodes as the dependent variable and SNR and group as the independent variables, as well as MWT-B score as a covariate. Finally, correlation analysis was conducted to assess a possible relation between the N1 and P2 amplitudes and PANSS scores in participants with SZ by determining the 2-tailed Pearson product–moment correlation between the corresponding amplitudes at electrode positions (Fc1, Fz, Fc2 and Cz) and PANSS scores separately for SNR conditions. For the analysis of electrophysiological data, 8 subjects had to be excluded (2 HC, 6 SZ) due to strong EEG artifacts.

Bonferroni-corrected post hoc paired-sample *t*-tests were calculated when appropriate. Whenever necessary, Greenhouse-Geisser-corrected *p*-values were applied. All tests were two-tailed and *p* values ≤ 0.05 were considered significant.

## 3. Results

### 3.1. Behavioral Results

**Figure 2:**
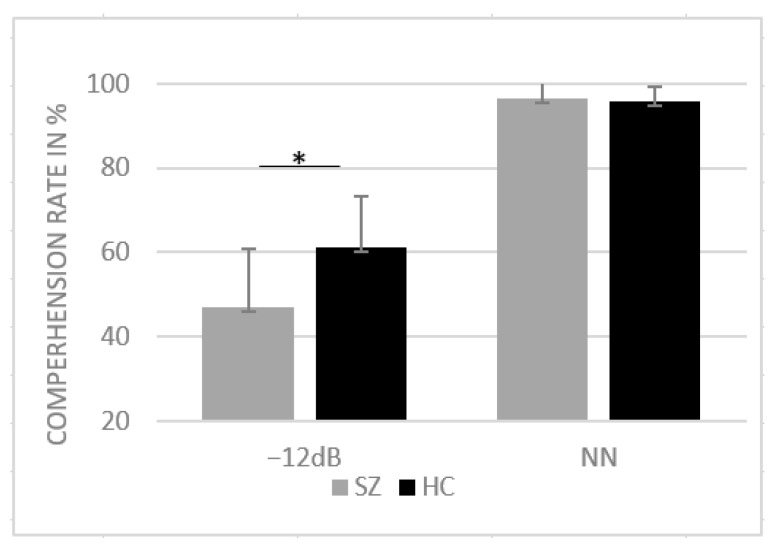
Mean comprehension rates in percentage and standard deviation for HCs and SZs for SNR conditions NN and −12 dB SNR. * *p* < 0.05.

For the correct responses, the ANOVA yielded a significant main effect of group (F(1, 39) = 10.47, *p* = 0.002, η^2^ = 0.21) as well as a significant interaction between SNR and group (F(1, 39) = 12.31, *p* < 0.001, η^2^ = 0.24). Post hoc paired-sample *t*-tests revealed a significant difference between groups for −12 dB SNR condition (t (39) = 3.50, *p* = 0.001, d = 13.02) with reduced comprehension in SZs compared to NN. For NN, the Shapiro–Wilk test revealed violation of normal distribution (*p* < 0.05); thus, the nonparametric Mann–Whitney U test was performed to assess group differences in the NN condition. No group differences were found in the NN condition (U = 191, z = −0.52, *p* = 0.599). Mean comprehension rates are provided in Figure 2.

### 3.2. Electrophysiological Results

**Figure 3:**
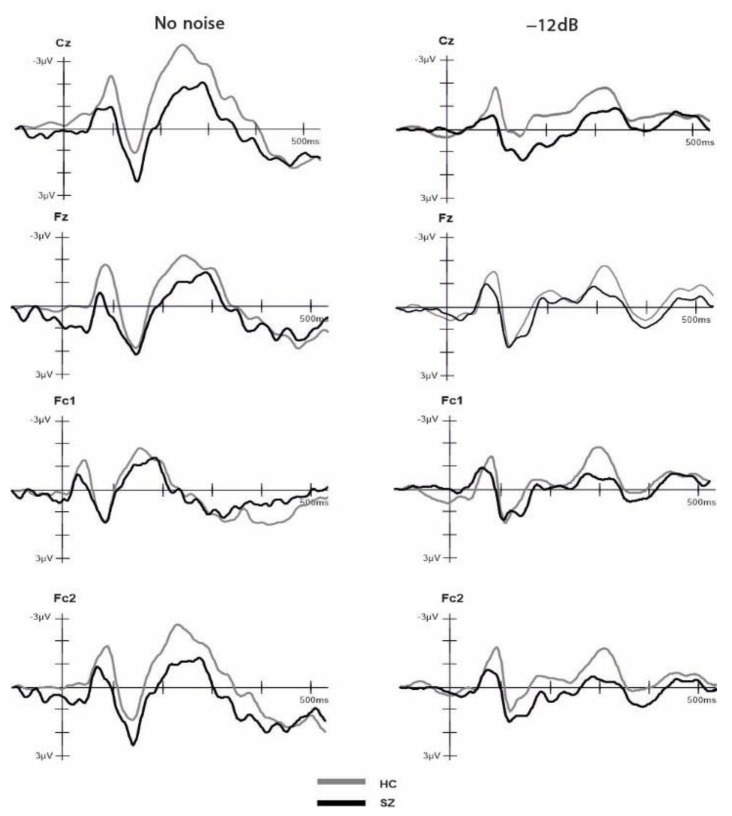
Event-related potentials (ERPs) time-locked to auditory onset at electrodes Cz, Fz, Fc1 and Fc2 for the healthy controls and schizophrenia groups. The ERPs are shown separately for −12 dB and no noise condition.

Two independent *t*-tests were performed to compare the number of included epochs in the analysis for each group and SNR. The results indicated no significant difference (*p* > 0.05) between the groups in both conditions, HC (NN: M = 18.42, SD = 1.74, Min = 14, Max = 20; −12 dB: M = 73.26, SD = 7.40, Min = 49, Max = 80); SZ (NN: M = 17.5, SD = 2.53, Min = 12, Max = 20; −12 dB: M = 68.71, SD = 10.45, Min = 49, Max = 80). The ANOVA comparing group, electrode and SNR condition for the P2 amplitude component did not yield any significant main effects nor interactions (all *p* > 0.05). The ANOVA comparing group, electrode and SNR condition for the N1 amplitude revealed a significant main effect of group (F(1,31) = 4.44, *p* = 0.043, η^2^ = 0.125), with more negative amplitudes (larger N1 component) for HCs (M = −2.26, SD = 1.16) compared to SZs (M = −1.40, SD = 1.16). Moreover, our analysis revealed a significant main effect of SNR on the N1 amplitude (F(1,31) = 4.726, *p* = 0.037, η^2^ = 0.132). Specifically, the N1 amplitude was more negative in the no-noise condition (M = −2.102, SD = 1.49) compared to the −12 dB condition (M = −1.552, SD = 1.27). Furthermore, we observed a significant interaction effect between electrode and SNR (F(1,31) = 2.733, *p* = 0.046, η^2^ = 0.082). Post hoc pairwise *t*-tests with Bonferroni adjustment showed that at the Cz electrode, the NN condition (M = −2.45, SD = 1.74) exhibited a significantly larger negative amplitude compared to the −12 dB condition (M = −1.53, SD = 1.56), t(32) = −3.444, *p* = 0.002. No other main effects nor interactions were significant (all *p* > 0.05). The ANOVA comparing group, electrode and SNR condition for the N1-P2 complex revealed a significant main effect of SNR on the N1-P2 complex (F(1,31) = 8.131, *p* = 0.008, η^2^ = 0.208). Specifically, we found a larger N1-P2 complex for the no-noise condition (M = 4.29, SD = 1.98) compared to the −12 dB SNR condition (M = 3.41, SD = 1.85). No other main effects or interactions reached statistical significance (all *p* > 0.05). To examine the impact of cognitive ability, as measured by the MWT-B, on the observed N1 differences, we conducted a factorial ANCOVA. The dependent variable was N1, while the independent variables were SNR and group. The MWT-B scores were incorporated as a covariate. The result indicated that, after controlling for MWT-B scores, the main effect of SNR was no longer statistically significant (F (1,26) = 0.12, *p* = 0.91). However, the main effect of groups retained its significance (F(1,26) = 9.653, *p* = 0.005, η^2^ = 0.271). Grand-average ERPs across groups in response to the NN and −12 SNR conditions are provided separately in Figure 3. The amplitude values of ERPs for each group and condition are shown in Table 2.

The Pearson correlation analysis indicated significant relationships between the positive subscale of the PANNS and N1 amplitudes at Fz (−12 dB: r (12) = 0.622, *p* = 0.017; NN: r(12) = 0.678, *p* = 0.008) and Fc2 (−12 dB: r(12) = 0.613, *p* = 0.020; NN: r(12) = 0.612, *p* = 0.020) in both conditions and a positive relationship between the positive subscale of the PANNS and N1 amplitudes at Fc1 only in the NN (r(12) = 0.712, *p* = 0.004).

## 4. Discussion

Impaired language function, such as disorganized speech, is a hallmark symptom of schizophrenia [45]. Recent studies have not provided conclusive evidence regarding the underlying neural mechanisms of audiovisual speech perception. Moreover, previous research has been limited by primarily focusing on late stimulus processing. The present study aimed to address this limitation, taking into account the importance of early neural speech processing [32]. To achieve this goal, the present study investigated the early processing of audiovisual speech perception in individuals with schizophrenia and healthy controls using a naturalistic speech-in-noise paradigm with bimodal (audiovisual) disyllabic nouns.

As expected, participants in both groups demonstrated impaired speech perception in the −12 dB condition compared to the NN condition, providing further evidence that speech perception is limited in noisy environments [9,43].

Our results demonstrate a similar performance for both groups in NN. However, it is noteworthy that the performance of both groups in NN reached ceiling level (~95%), which makes it difficult to draw precise conclusions regarding potential group differences. Nonetheless, group differences were more pronounced in the −12 dB condition: SZ patients showed a larger impairment in word recognition compared to healthy participants.

Research has shown that healthy participants benefit most from the presentation of visual articulation at a SNR of −12 dB, compared to other intensities of white noise. Liu et al. [43] and Ross et al. [9] compared the audiovisual speech perception in healthy participants across different SNRs and found the highest multisensory gain at a SNR of −12 dB. In a subsequent study, Ross et al. [39] demonstrated the largest group difference between HCs and SZs at the −12 dB SNR condition, with less audiovisual enhancement in SZs. These findings are in line with our results that also show a reduced speech perception in SZs in −12 dB SNR.

Alongside the existing evidence suggesting deficits in multisensory speech perception in SZs [22,24,27,28,34], these patients also show deficits in unisensory visual [46,47] and auditory processing [48,49,50,51,52,53], which can affect speech comprehension independently. In addition to the reported behavioral data, the electrophysiological results may offer further insights into the observed group difference.

Studies employing an additive model approach to investigate the integration of audiovisual speech consistently demonstrate attenuated auditory N1 and P2 components compared to unimodal processing of stimuli [3,32,34]. However, there is a limited and inconsistent body of literature regarding the difference between SZs and HCs specifically concerning the P2 component during multisensory speech processing. In the present study, we observed no group differences in the P2 component, which aligns with the findings of Stekelenburg et al. [34], where no P2 difference was reported between HCs and SZs in the audiovisual speech condition. However, Senkowski and Moran [35], in their investigation using speech syllables with varying SNRs, found reduced P2 amplitudes in SZs, although this reduction was independent of the noise level. One possible explanation for the lack of P2 suppression in our study may be related to the group differences in the N1 component, which was significantly reduced in SZs compared to HCs. Previous studies reported similar findings of reduced N1 amplitudes in SZs for both non-speech stimuli [54,55] and speech stimuli: Senkowski and Moran [35] found reduced N1 amplitudes in SZs compared to controls when presenting single syllables with additional information about lip movements. This difference in N1 amplitude was only present in the no-noise condition and diminished in the low- and high-noise conditions. However, we could not replicate this finding, as we did not observe any significant group differences as a function of SNR. Furthermore, in terms of behavioral results, Senkowski and Moran [35] observed the most pronounced group difference in the NN condition. In contrast to this, and consistent with the findings of Ross et al. [39], we observed group differences only in the −12 dB SNR condition. This difference between the studies seems to be related to the different attentional demands induced by the tasks, as Senkowski and Moran [36] only presented single syllables instead of meaningful words. These differences in attentional demands are also reflected in different hit-rates in the NN condition, with Ross et al. [39] and our study reporting a hit-rate at ceiling level (more than 90%) for both groups and Senkowski and Moran [35] reporting only 80% for HCs and 57% for SZs. Moreover, providing further support to this notion, we initially observed a significant reduction in N1 amplitude in the −12 dB SNR compared to the NN condition. However, this difference diminished after controlling for the participants’ cognitive ability. Existing research emphasizes the critical role of attention in the modulation of integrative sensory processing [56,57]. Therefore, a possible explanation for our result may be reduced attention in SZs, as selective attention allocation on a sensory processing stream leads to a decrease in the N1 component [58,59]. This interpretation is also consistent with well-known cognitive deficits described in SZs [60,61,62,63]. Additionally, the N1 component is also documented to be linked with auditory processing [59,64]. Salisbury at al. [54] associated a reduction in the N1 component in SZs with abnormalities of the primary N1 generators in the superior temporal gyrus and therefore the primary auditory cortex. Accordingly, it is reasonable to assume that deficits in speech perception in SZs may be a result of deficient auditory processing [35]. In line with this assumption, we did not find any significant group differences for the N1-P2 complex. The N1-P2 complex is a well-documented correlate of audiovisual speech perception with reduced amplitudes for audiovisual compared to unisensory speech presentation [29,64,65,66]. Therefore, one would expect group differences between SZs and HCs in the N1-P2 complex if deficits in audiovisual integration were crucial for the observed deficits in speech perception in SZs. Furthermore, we found that the reduction in fronto-central N1 amplitudes correlates with more positive symptoms (i.e., hallucination, delusion, disorganized thinking) as measured with the positive subscale of the PANSS. This finding is consistent with prior research indicating that a reduced auditory N1 amplitude serves as an endophenotype of SZ [54,67]. In a study investigating individuals that experienced their first episode of schizophrenia (*n* = 71 hallucinators, *n* = 27 non-hallucinators), it was found that hallucinators had reduced N1 amplitudes compared to non-hallucinators and healthy individuals [68]. These results provide support for the hypothesis that hallucination may stem from auditory cortex dysfunction, indicating that auditory ERPs might be a potential neurophysiological endophenotype for SZ [68].

A potential confounding factor that should be carefully considered while examining N1 in SZs is antipsychotic medication [69]. The question of whether N1 reductions are primarily caused by medication or by the underlying pathology of the disease has been explored in various studies, yielding inconclusive findings [70,71,72,73]. However, recent genetic investigations focusing on N1 in twins who are either concordant or discordant for schizophrenia [74] and unaffected family members [75] provide persuasive evidence that N1 reduction is related to inherited abnormalities in cortical processing in SZs. All in all, the present findings implicate an influence of unisensory auditory processing on multimodal speech perception.

## 5. Limitations

The present study contributes to the understanding of audiovisual speech perception in SZs by highlighting neurophysiological correlates of speech perception under naturalistic conditions. However, there are several limitations that should be addressed. First, our study design did not entail unisensory conditions, which makes it challenging to determine the specific impact of audiovisual integration deficits on speech perception. Future research should consider incorporating unisensory conditions to better understand the role of audiovisual integration in SZs.

Given the heterogeneity of symptoms in SZs and previous findings suggesting a relationship between specific symptoms such as auditory hallucination and impairment in auditory perception [49], it might be fruitful for future studies to investigate audiovisual integration within different schizophrenia subtypes. Additionally, considering the influence of dopamine on basic mechanisms of multisensory integration, such as the temporal and spatial binding window [76], further research is needed to explore the effect of medication on audiovisual speech perception. Lastly, even though the sample size was sufficient to answer the research questions, a larger sample would enhance the generalizability of the presented results.

## 6. Conclusions

In natural conversational situations, speech comprehension often occurs in the presence of background noise. Visual cues, such as lip movement, can be beneficial in improving our speech comprehension in noisy environment. The present study highlights that patients with schizophrenia show impairments in speech perception under noisy environmental conditions. Moreover, we found altered N1 amplitudes in SZs during speech perception, while P2 amplitudes, as well as the N1-P2 complex, were similar to those of HCs. These findings suggest that multimodal speech perception may be disturbed at an early stage of speech processing and may be due to deficits in auditory speech perception. Further research including unisensory conditions is necessary to extend the present findings on neural correlates of audiovisual speech perception in SZs.

## Figures and Tables

**Table 1:** Sample characteristics for SZs and healthy controls (HCs).

	SZs (N = 20)		HCs (N = 21)		Significance
Characteristics	M	SD	M	SD	
N (female, male)	20 (8, 12)		21 (9, 12)		X^2^(1) = 0.34, *p* = 0.85
Age in years	41.9	13.8	38.2	13.0	t(39) = −0.85, *p* = 0.95
Years of education	10.7 (N = 19)	1.7	12.4 (N = 18)	1.2	t(35) = 3.61, *p* = 0.08
MWT-B	98.6 (N = 15)	5.3	106.6 (N = 19)	4.7	t(32) = 4.6, *p* < 0.001
PANSS (total)	49.5 (N = 18)	10.0			
Positive	11.5 (N = 18)	3.9			
Negative	12.2 (N = 18)	4.8			
Illness years	13.9 (N = 17)	10.2			

**Table 2:** Amplitude values of N1, P2 and N1-P2 for SZs and HCs at SNR conditions NN and −12 dB.

	SZs (N = 14)		HCs (N = 19)	
	NN	−12 dB	NN	−12 dB
Amplitude	M (SD)	M (SD)	M (SD)	M (SD)
N1	−1.54 (1.46)	−1.29 (1.41)	−2.66 (1.46)	−1.86 (1.26)
P2	2.45 (2.0)	2.09 (1.51)	1.92 (2.0)	1.62 (1.51)
N1-P2	3.99 (1.96)	3.34 (1.82)	4.58 (1.96)	3.47 (1.83)

## Data Availability

Research data were generated at Hannover Medical School and are stored on a hard drive therein. All data, including raw electrophysiological data, are available from the corresponding author.
